# Medico-Chirurgical Transactions

**Published:** 1845-01

**Authors:** 


### THE
### MEDICO-CHIRURGICAL
### REVIEW.

JANUARY, 1845.

### Medico-Chirurgical Transactions, published by the Royal Medical and Chirurgical Society of London. Volume the Twenty-seventh (the Ninth of the Second Series), 1844.

In the present dearth of medical works of sterling merit and original views, the appearance of the annual volume of the Royal Medical and Chirurgical Society is an event of some importance in medical literature. There have been few men of eminence in the profession in the present century who have not been contributors to these Transactions. Those long established in practice have enriched the pages of this work with the results of their matured views of disease, and with the curiosities of their medical experience, whilst the young and enterprising have made them a record of original inquiries and interesting observations in the ample field of pathological science. It must be confessed that the novelty of the volume, though not its substantial value, is in some degree lessened by the authorized publication in the weekly periodicals of an abstract of the papers read at the Society's meetings, and even of papers of which the titles only had been announced, owing to the press of communications sent in at the close of the season. For this reason, we think that the publication of the present volume should not have been delayed to so late a period as the month of November. The papers are communicated to the Society in a state ready for the press, and the task of selection—of winnowing the grain from the chaff, may be said to commence at the beginning of the session. We are aware of one alleged cause of delay, viz. the time occupied in colouring the plates. They are, however, too few in number (only three) to account for the late period at which the volume has this year been delivered to the members. We are glad, however, to see some coloured illustrations. Pathological appearances cannot always be satisfactorily represented by plain lithographs, and the Society is sufficiently prosperous to afford the additional expense of colouring them. The present number is thicker than any of the later volumes, having no less than five hundred pages, and as usual contains many valuable communications to which we shall now direct the attention of our readers.*

* The Council have done well in placing round the Meeting-room the busts of

### I. An Account of Two Cases of Rupture of the Ureter, or Pelvis of the Kidney, from External Violence, followed by large Effusion of Urine into the Abdomen. By *Edward Stanley*, F.R.S.

Cases of rupture of the urinary bladder by external violence are by no means of rare occurrence, and as the laceration usually involves that portion of the viscus which is invested with serous membrane, the urine escapes into the sac of the peritoneum and gives rise to fatal peritonitis. But the pelves of the kidneys and the ureters are differently situated, so that a rupture of their walls would only permit the escape of the secretion of the kidneys into the surrounding cellular tissue, where we can easily understand that actions might be set up to limit the diffusion of the fluid and to avert the dangerous consequences of the injury. It may also be observed, that these parts are by their position well protected from injuries, and being channels for the transmission of the urine, and not organs for its retention, they are less liable to be ruptured by external violence than a distended urinary bladder, which sufficiently explains the rarity of the accident described by the President in the two interesting cases which he has communicated to the Society.

A boy, aged 9 years, was brought to St. Bartholomew's Hospital, having been squeezed between the wheel of a cart and a curb-stone.

"The immediate consequences were, severe contusion of the soft parts around the pelvis, inability to walk, and great pain in the lower part of the abdomen; he lay helpless, apparently suffering from severe internal injury. Much ecchymosis ensued in the integuments around the pelvis, and extensive suppuration in the subcutaneous cellular tissue, from which several ounces of matter were discharged by puncture near the left sacro-iliac symphysis. By the end of the sixth week, recovery of the injured soft parts around the pelvis had considerably advanced. At this period, my attention was directed to a fulness not before observed on the right side of the abdomen, and on further examination, a circumscribed, oblong swelling was recognized through the abdominal parietes, extending from the base of the chest downwards to within a short distance of Poupart's ligament; anteriorly, it terminated abruptly at the linea alba; posteriorly, it could be traced into the lumbar region, but it here presented no distinct boundary; the liver appeared to be pressed upwards by the swelling, so that the right lung did not extend below the sixth rib, the admission of air into it here ceasing in a defined line. Pressure on the swelling gave no pain, but a deep fluctuation in it could be recognised. The urine passed naturally, as it had done throughout, and that there was no distension of the bladder was ascertained by the introduction of a catheter. I could but suppose the swelling to be an abscess, but there was difficulty in adopting this opinion of it from the absence of pain and constitutional derangement. To discover the nature of the swelling, I made a small puncture in it with a lancet, from which a little clear yellow fluid escaped; this was followed by much pain in the abdomen, which yielded to the application of leeches. By this exploratory proceeding, I learned that the fluid was situated immediately beneath the abdominal muscles, and that it was not pus. Three

---

some of the more distinguished Members and former Presidents of the Society. They form elegant and appropriate ornaments to the walls, and Halford, Cline, Babington, Cooper, Abernethy, Travers, Earle and Brodie have well deserved the honour awarded them.

weeks afterwards, the abdominal swelling having become more tense and pointed, I punctured it with a small trochar midway between the last rib and crista of the ilium, and discharged from the opening fifty-one ounces of a clear yellow fluid. Pain in the abdomen followed this puncture, but it yielded as before to the application of leeches. Eleven days afterwards, the swelling having again enlarged, I discharged from it by puncture fifty-eight ounces of a clear yellow fluid. In sixteen days from this puncture, the swelling having again greatly increased, I removed from it sixty-four ounces of fluid. The swelling returned, and having acquired a certain size, it remained stationary, on which account it was not interfered with for nearly three months, when I again punctured it, and discharged seventy-two ounces of fluid of the same characters as before. Three weeks afterwards, I punctured the swelling the sixth and last time, but discharged only six ounces of fluid, and it appeared to me that a larger quantity was not obtained from the existence of some obstacle to the canula fairly entering the cavity in which the fluid was contained. The other parts of the treatment consisted in the repeated applications of leeches, in the application, on one occasion, of a large blister to the swelling, and in the use for a considerable time of the ointment of iodide of potassium: but, with the exception of the leeches, which relieved the pain in the abdomen recurring more or less after each puncture, it is doubtful whether the other measures were of any service. Throughout, the general health had been good, and all the functions of the body appeared to be perfectly performed. From this period the swelling continued without increase or obvious diminution: it still extended from the linea alba into the right lumbar region, and as any further interference by operation or otherwise, was now considered inexpedient, I discharged the boy from the hospital nine months after the occurrence of the accident. At several subsequent periods I have seen him in good health, with the abdominal swelling still distinct, but, as we have thought, slowly diminishing, and with less evident fluctuation." 5.

Mr. Stanley remarks that three questions of interest here arise—what was the nature of the fluid so largely accumulated in the abdomen; where was it situated; and, if it was urine, from what part of the urinary apparatus was it derived? The first question is answered by the chemical examination of the fluid evacuated made by Mr. Edward Ormerod and Mr. Taylor, who detected, in addition to a large quantity of urea, the other ordinary ingredients of urine. Mr. Taylor further observed, that from the absence of mucus in the fluid, the probable source was high in the urinary apparatus, as at the commencement of the ureter. With respect to the situation of the fluid, Mr. Stanley discusses the question, whether it was lodged within the peritoneal sac, or had formed for itself a cavity by detaching the peritoneum from the abdominal and lumbar muscles, and from the circumscribed character of the swelling, and especially its abrupt termination at the linea alba, where its further progress would be impeded by the firm connection of the peritoneum with the abdominal aponeuroses, as well as from the absence of peritoneal inflammation, and the formation of the tumor without pain or constitutional derangement, we think justly concludes the latter to be the situation where the fluid had accumulated.

The second case also occurred in St. Bartholomew's Hospital, under the care of Mr. Vincent. A woman was admitted immediately after having been knocked down and, as it was stated, pushed some way before the wheel of a cart. The left femur was broken and she was much hurt in the left hypochondrium. On the following day there was much febrile disorder, and severe pain and distension of the abdomen. After bleeding from the arm, the application of leeches to the abdomen, and the administration of calomel and antimony, the general distension and pain subsided, but there remained a circumscribed and painful swelling in the right hypochondrium. It increased and formed a fluctuating tumor which was supposed to be an abscess probably connected with the liver. Mr. Vincent punctured the swelling with a small trocar and discharged between two and three pints of a straw-coloured urinous-smelling fluid. The patient was much relieved; but in about ten days the fluid had again accumulated so as to occasion much distress. Six pints of a similar fluid were again removed, with temporary relief. But the swelling returned and she gradually sunk and died in the tenth week from the receipt of the injury. The fluid obtained from the abdomen was found to be albuminous, and to contain a small quantity of urea.

"Upon examination of the body, a large cyst was found on the right side of the abdomen behind the peritoneum extending upwards to the diaphragm, and downwards to the pelvis. The boundaries of this cyst were formed by lymph and thickened cellular tissue; within it was a large quantity of fluid presenting the characters of a mixture of pus and fætid urine. A passage was found extending from the upper part of the cyst into the pelvis of the right kidney. The aperture in the pelvis of the kidney was large and irregular; the appearances were such as might be expected to result from laceration of the membranous structure composing it. The liver presented in its anterior border, the marks of a slight laceration of its tissue, which was in progress of healing. The kidneys were slightly granular." 10.

Mr. Stanley notices a circumstance exhibited by these cases which may be of importance in practice, viz. the absence of any symptom of immediate occurrence leading to a suspicion of injury to any part of the urinary apparatus, and concludes by suggesting the question, whether the best proceeding would be gradually to withdraw the fluid by repeated punctures of the cyst, and thus to favour the collapse and adhesion of its sides upon the plan recommended by Mr. Abernethy for the treatment of lumbar abscess, or whether the urinary cyst should be punctured at its lowest part, in the view of maintaining the aperture free for some time, that the fluid may drain from it, upon the plan which has been adopted in cases of empyema for the discharge of fluid from the chest. We should be inclined to give the preference to the plan adopted by Mr. Stanley and Mr. Vincent in these cases, because we conceive that a large cyst containing urine mixed with morbid secretions would, if left with an open communication, be as liable to inflame and to give rise to severe constitutional derangement and hectic symptoms as the cyst of a large lumbar abscess.

### II. ACCOUNT OF A CASE OF CYSTICERCUS CELLULOSÆ OF THE BRAIN. By *Drewry Ottley*, Esq.

This is a form of hydatid which sometimes occurs in large numbers in the brain, especially of persons dying at an advanced age. Cruveilhier observed in the brain of an epileptic patient who died in the Bicêtre at Paris at least a hundred of these parasites. Some were seated in the subarachnoid cellular tissue of the brain and cerebellum; others occupied the substance of the convolutions and central part of the hemispheres, and as many as fifty were detected in the structure of the cerebellum. In the case related by Mr. Ottley we have a better account of the disorder of the cerebral functions produced by these bodies than has generally been given by those pathologists who have discovered them. The patient was the wife of a chimney-sweeper about forty years of age. Cerebral symptoms first appeared in the early part of 1838 and chiefly consisted of frequent giddiness, dull pain in the head, loss of memory and confusion of intellect. In 1839 she became subject to fits, during which there was entire loss of consciousness with convulsions of the limbs.

"The character of these attacks was different from that of ordinary epileptic fits; they were less sudden, both in their invasion and their termination, and the convulsions ceased and recurred as often as eight or ten times in as many hours, the stupor remaining during the intervals, and after their cessation. The recovery from them was slower also, for she would often remain for two or three days in a stupified state, with difficulty roused to understand a question, and incapable of replying to it, sometimes using wrong words, at others unable to pronounce the words she wished to employ.

"During the last twelvemonths of her life, her sufferings became more constant. She could not venture to walk alone, for she would stagger as she went, or suddenly become so confused as not to know in what street she was walking; and more than once she was attacked with a fit in the streets. The pain in the head was now constant, though never *extremely* acute; her sight became dim; the convulsive attacks recurred more frequently; and, at length, towards the end of October 1840, after being frightfully convulsed for twenty-four hours, with the face twisted over the right shoulder, and the pulse becoming more frequent and feeble, she expired, without having had any return of consciousness." 14.

On examination of the body,

"The vessels on the surface of the brain were found moderately congested, and the sub-arachnoid cellular tissue was infiltrated with serum; but the most remarkable morbid appearance which the organ exhibited arose from the presence of numerous small fibrous cysts in the pia mater, covering the surface of the hemispheres, and dipping between the convolutions of the brain. These cysts were present on both sides, but were most numerous on the surface of the left hemisphere. They varied in size from that of a pea to that of a small peppercorn; they were seated in the pia mater, but had become partially imbedded in the grey matter of the convolutions. None existed in the white matter, in the central ganglia, nor in the plexus choroïdes. A few were found at the under surface of the cerebral convolutions; but none either in the cerebellum or medulla oblongata. The cerebral tissue around the cysts appeared natural, as to colour and consistence, and the brain generally, except for the presence of these animals, would have been termed healthy. There was, I should add, however, rather more fluid in the ventricles, and at the base of the brain, than is natural."

The cysts were found to contain vesicular entozoa, presenting all the characters of the cysticercus cellulosæ. In some of the cysts the animal had evidently perished, and was undergoing decay.

### III. On the Cause of the Occasional Presence of Spermatozoa in the Fluid drawn from the Sac of common Hydrocele of the Tunica Vaginalis. By *John Dalrymple*, Esq.

We were disappointed on reading this paper by so able a pathologist as the author, to find that it adds no new fact, and throws no new light on the subject of the presence of spermatozoa in the sac of the tunica vaginalis.

Mr. Dalrymple gives some extracts from the writings of Scarpa on the altered relative position of the vessels of the spermatic cord in scrotal hernia and in hydrocele, which we differ from him in believing to be known to all well-informed surgeons, and he adds the particulars of a dissection of the parts in a common hydrocele to illustrate some anatomical points, which scarcely needed further confirmation. Mr. Dalrymple's object is to show that the epididymis and vas deferens are placed by no means out of danger of being punctured by the trocar, and that the wounding of a seminal tube might afford the few spermatozoa occasionally found in common hydrocele, but in the solitary case upon which his observations are founded, no spermatozoa were discovered in the sac of the tunica vaginalis, nor does it appear that either the epididymis or vas deferens had been wounded with the trocar, and the probability of the supposition as to the source of these bodies adopted by the author, is lessened by the fact that they have been found after death in a tunica vaginalis which had never been submitted to an operation. In consequence of the vas deferens and epididymis being displaced towards the external part of the sac in cases of hydrocele, Mr. Dalrymple is of opinion—

"That the position at which the trochar should be entered, ought to be made much more towards the mesial line than is usually adopted, and that the antero-lateral or antero-external aspect should in future be avoided, unless after the most careful and accurate examination the true position of the parts should be determined." 24.

The displacement is not in general of sufficient extent to render this caution necessary, and we believe that the rules commonly given for the performance of the operation of tapping a hydrocele may continue to be safely acted upon.

The next paper is not in the order of succession but relates to the same subject as the preceding one.

### IV. AN ACCOUNT OF THE EXAMINATION OF A CYST CONTAINING SEMINAL FLUID. By *James Paget*, F.R.C.S.

The author, believing that the presence of spermatozoa in the fluids of certain hydroceles has not yet been illustrated by examinations after death, contributes the following observations:—

"A middle-aged man was admitted into St. Bartholomew's Hospital, under Mr. Stanley, six months ago, with what was regarded a common hydrocele of the tunica vaginalis on the left side. This was tapped, and several ounces of a serous fluid, of the kind usually found in such hydroceles, were drawn off. The fluid was not particularly examined; the hydrocele was not injected; and, soon after, the man left the hospital. He returned with the hydrocele again full; and, besides, with very extensive abscesses in the perineum and fistulous openings into the urethra and bladder. With these he died, extremely emaciated, on Wednesday, June 5th; and the parts connected with the hydrocele were removed for examination.

"The sac which had been tapped, and which, in its external appearance, even after its removal from the body, had all the characters of a common hydrocele, was found to be completely separate, and its cavity distinct from that of the tunica vaginalis. It was situated just above the testicle, and in front of the spermatic cord; it was of an elongated oval form, four inches in length, and held about six ounces of a bright light-yellow fluid, containing a small quantity of albumen, but no trace of either spermatozoa or any other organic particles. Its walls were very thin, and loosely connected with the adjacent parts; they were composed of well-organised delicate fibro-cellular tissue, and their internal surface was smooth, but not lined by any epithelium. The sac was closed on every side: it was separated from the tunica vaginalis by tissue like that of false membrane, layers of which formed several incomplete cysts, or spaces, containing a serous fluid like that in the sac itself." 399.

The tunica vaginalis appeared healthy and contained three drachms of fluid similar to that in the sac, and also destitute of any trace of spermatozoa or any other constituent of the semen.

"By the upper part of the epididymis, on its inner side, and attached to its surface, where on each side the tunica vaginalis is reflected from it, there was a globular cyst, about two-thirds of an inch in diameter, completely distinct from those already described, though almost surrounded by them. Its walls were thin, but opaque white; they were composed of fibro-cellular tissue, with delicate pale filaments, intricately interwoven, and not so fully organized as that of the large sac. From its polished inner surface I scraped scales of an exceedingly delicate tessellated epithelium, composed of very pale, elongated, oval, and angular cells, united by their obscure edges, and having dark large nuclei of the same shape as themselves: they were much like the epithelium-cells of the tunica vaginalis itself, but even smaller than those of the blood-vessels. The contents of the cyst were two or three drachms of an opaque whitish fluid, in which there were numerous spermatozoa, dead and small, but well formed, and still more numerous granules, and large round granular spermatic globules. It contained no albumen coagulable by heat.

"This cyst was closed on every side, and loosely connected to the adjacent parts, so that without cutting, and with very little force, it could be easily separated from them. The part of the surface of the epididymis to which it was attached was left, after its separation, perfectly smooth, and without the least appearance of a breach in the investing membrane, beneath which the fine convolutions of the seminal duct were seen, uninjured and undisturbed. It was as evident as it could be that the cavity of the cyst was completely isolated from every part of the seminal tubes." 401.

These cysts appear to correspond with those serous sacs minutely described by Mr. Curling in his recent work on the Testis, under the head of Encysted Hydrocele of the Epididymis, and stated to be of very common occurrence. Mr. Paget remarks:—

"If, with the aid of these observations, we endeavour to find an explanation of the occurrence of spermatozoa in the fluid of cysts connected with the testicle, we may suppose either that the fluid part of the semen has permeated from the seminal tubes into the cysts, and been further organized in them; or, that the cyst itself secretes a fluid in which the organic structures of the semen may be developed. Such a permeation is hardly possible; for the fluid would have to pass not only through the vascular wall of the tubes, but through two or three more layers of vascular tissue, by all of which it would be absorbed rather than transmitted. The most probable explanation of these cases, therefore, seems to be, that certain cysts, seated near the organ which naturally secretes the materials for semen, may possess a power of secreting a similar fluid. And this explanation is in some measure supported by the analogy of those cysts which are found in the ovaries, and more rarely in other parts of the body, especially beneath hairy parts of the skin, and in which the ordinary products of the skin, such as epidermis, sebaceous matter, hair, &c., are formed on the genuine cutaneous tissue of their internal surface." 402.

Mr. Paget's explanation of the vicarious appearance of the spermatozoa, which has of late so much puzzled the members of the Society, has the merit of being ingenious and original, though it must be added that we are by no means satisfied with it. There is considerable doubt whether one secreting organ, or part, can completely perform the offices of another of different structure, or wherefore the necessity for the variety of tissue and complicated arrangement of the glands, and it must be observed that the spermatozoa found in some of the recorded cases of hydrocele have appeared as perfect and as well developed as any taken from the vas deferens or vesiculæ seminales. The cause of the presence of these bodies in the different forms of hydrocele still requires further investigation.

### V. Cases of Carcinoma of the Thyroid Gland. By *Cæsar Hawkins*, Esq.

This is a disease generally regarded by writers as of rare occurrence, but Mr. Hawkins is inclined to believe that the thyroid gland may be more often the seat of primary scirrhus than is usually supposed. He first gives the case of an old man who was admitted into St. George's Hospital on account of a large and hard tumor occupying the whole of the anterior part of the neck and impeding respiration, which Mr. Hawkins conjectures, and perhaps rightly, to be a case of the disease alluded to; but the patient subsequently left the hospital to go into the country and was lost sight of. In a second case the history is complete.

"Thomas Holder, æt. 50, was admitted into St. George's Hospital May 17th, 1843, having the appearance of perfect health, with a considerable enlargement of the whole thyroid gland, but particularly of the right lobe, which projected upwards more than the left; the tumor was uniformly smooth on the surface, and very firm and solid; it was completely fixed to the larynx, and sufficiently free from attachment to other parts to move with all the motions of the larynx and œsophagus; the skin was unattached and unaltered in colour, and the superficial veins were large. He breathed with some noise, and had a slight cough, but could respire naturally when told to do so. Deglutition was performed with somewhat more difficulty than respiration, although the larynx and trachea were thrown very much to the left side of the neck, nearly two inches perhaps out of the central line. The tumor was free from pain and tenderness. The patient was deaf and dumb, so that a full history could not be obtained at first; but it was learned that the first appearance of the tumor was only about five weeks before his admission." 29.

The nature of the tumor was concluded to be carcinomatous, and the case was treated with iodide of potassium, which was employed both internally and locally, but without benefit. The tumor increased in size, and on the 13th of July was perhaps half as large again as on his admission. He became considerably emaciated and died on the 23rd. He suffered severely from dysphagia, which prevented any solid food from being taken, and even liquids were swallowed with difficulty, so that he frequently was obliged to lean on the table from threatened suffocation in the act of eating, and from vomiting, which sometimes occurred regularly at a certain period after eating, and at other times took place violently during his meal. He also suffered much from pain in the epigastrium and hypochondria, and had tenderness over the stomach, while the respiration seemed little interfered with. About the middle of July, the pain being then much increased, he began to vomit some coagula of blood, but this again lessened while he was taking some lead and opium. For a few hours before death he had much difficulty of breathing. The following is an account of the *post-mortem* examination.

"Upon the anterior surface of the windpipe was a large tumor, which extended from the thyroid cartilage to the sternum. Laterally it projected beyond the sterno-mastoid muscles, the fibres of which, as well as those of the sterno-hyoid, omo-hyoid, and sterno-thyroid muscles of both sides, were expanded over, and partly imbedded in the tumor. The right internal jugular vein, common carotid artery, and pneumogastric nerve, were separated from each other by the pressure of the morbid growth. The vein was closely adherent to the tumor, and its coats, in one place, had been absorbed, and a soft part of the tumor projected into its interior, and a large clot of blood was firmly attached at this part. The artery was deeply imbedded in the tumor, and the pneumogastric nerve much flattened, and its fibrils separated so as to present a plexiform appearance.

"The thyroid gland had nearly disappeared, the only part which was left being a small portion of the left lobe intimately joined to the tumor, so as to show that they were originally portions of the same body, and this portion that remained was perfectly natural, and there was a complete line of demarcation between the two structures. The anterior surface of the windpipe was healthy.

"Posteriorly the morbid growth had extended to the pharynx and œsophagus, and to the cellular tissue connecting them with the larynx and trachea. The posterior part of the œsophagus was healthy, but the anterior part presented a large, irregular, ulcerated mass, extending from the arytæno-epiglottic ligaments to the first three or four rings of the trachea, and projecting into the interior of the pharynx and œsophagus, its surface being of a dark green colour, and covered with shreds and portions of sloughs, which were very fætid. The larynx and trachea had been quite pushed over to the left side, forming a curved line, and the right arytæno-epiglottic ligament was much thickened and altered in texture, and immediately below the cricoid cartilage a large ulcerated opening led into the trachea.

"Externally, the tumor presented an irregular lobulated appearance, the greater part being situated on the right side; internally, it presented the structure of genuine scirrhus; it was remarkably firm, in some places of a light yellow colour, and in others of a pale red tinge; the variety of scirrhus which it most resembled being the solanoid form; in fact, the section was very like that of a red potatoe.

"There were many small encephaloid tubercles at the base of both lungs, and in the cellular tissue under the costal pleura.

"The brain was wet, and the bloody puncta large, and the veins and sinuses were gorged with dark-coloured blood.

"The stomach was perfectly healthy, and rather small; the liver healthy, with a small serous cyst on the surface of the right lobe; the other viscera were healthy." 33.

Mr. Hawkins makes some sensible remarks upon the symptoms of this very interesting case.

"The symptoms enumerated, as caused by this tumor, included tenderness and pain in the epigastrium, vomiting after meals, and latterly hæmatemesis, which naturally led my attention to the stomach. During the first few weeks, careful examination detecting no hardness or swelling, and the symptoms not being constantly present, I did not think there was cancer of that organ; but during the last few days, the repeated vomiting of blood, and the great pain and tenderness of the epigastrium, and in that region only, made me express an opinion that there was most probably cancer of the stomach. The difficulty of swallowing, and the vomiting of blood were, however, satisfactorily accounted for by the ulceration and sloughing of the œsophagus and pharynx; but whence arose the marked pain and tenderness exactly over the stomach; which were more complained of by the patient than any other symptom, and existed even at the times when there was a temporary cessation of nausea?

"Looking to the highly expanded state of the fibres of the right pneumogastric nerve, is it not very probable that the symptoms in question depended upon this circumstance, the pain being referred (as with pressure on the spinal nerves) to the part where the nerves are finally distributed below the seat of pressure and irritation, which would in this case be chiefly the pyloric end of the stomach?

"I think I never saw the larynx so much turned out of its natural course by any tumor, as in this case, and dissection showed us also a cancerous degeneration of the side of the rima glottidis, and an ulcerated opening into the trachea; but yet the difficulty of respiration was never urgent till just before his death, and cough was very little complained of throughout the whole illness. He could always expand the chest freely, and without pain, and the stethoscopic signs showed only bronchitic effusion at the times that the obstruction about the glottis was more marked than usual. In fact, where there has been no pleuritic effusion, and no scirrhous alteration of the pleura, the little distress occasioned by encephaloid tubercles scattered through the parenchyma of the lungs is often very remarkable." 35.

The paper is concluded by some observations on the comparative frequency of scirrhus and encephaloid disease of the thyroid gland. The following is an appendix to the foregoing paper.

### Case of Scirrhus of the Thyroid Gland. By *R. Wilson Brown*, Esq.

Mr. G——, aged 60, of active habits and previous good health, began, in December 1842, to suffer from uneasiness about the larynx and slight cough and hoarseness, all which symptoms became aggravated before Christmas. "A hard swelling presented itself in the situation of the left lobe of the thyroid gland. The swelling was not prominent nor well-defined externally, but appeared to extend internally, and press upon the œsophagus, causing great uneasiness, and great difficulty of deglutition. Mr. G. remarked that his throat seemed to be bound, as it were, with an iron hoop. The integuments in the neighbourhood became thickly studded with hard tubercles of a cancerous character.

"Internally the disease continued to make progress, the difficulty of deglutition increased, the cough became more violent, convulsive, and almost incessant, with copious muco-purulent expectoration, streaked with scarlet blood."

He died in June 1843, after much suffering. The body was examined thirty-eight hours after death.

"The emaciation was very considerable. Numerous hard tubercles were seated in the skin, covering the throat and part of the chest and abdomen. The seat of the principal disease was in the left lobe of the thyroid gland, which was somewhat enlarged, and converted into a mass of carcinomatous structure, white, hard as cartilage, and with some gritty particles dispersed through it. The chain of lymphatic glands in the neighbourhood, on both sides, had undergone a similar change, and by these the œsophagus was compressed and reduced in diameter, and the larynx so firmly fixed in its situation, as to prevent any change of its position.

"There was not any ulceration of the inner surface of the œsophagus, nor any communication between it and the trachea. There was some thickening of the epiglottis, but not any disease of the trachea." 39.

Hard carcinomatous tubercles were also found in the lungs, and liver, in the glands surrounding the stomach and in the skin on the surface of the chest and abdomen.

So few cases of carcinoma originating in the thyroid gland are on record, that these cases are valuable additions to the history of cancer. The disease is not likely to be mistaken for ordinary bronchocele, which is a complaint of early life, whereas carcinoma is usually developed in persons somewhat advanced in age, according to Mr. Hawkins from the period of forty-five to sixty-five. The tumor too is harder, and has a more irregular surface than a bronchocele, and also makes more rapid progress. Mr. Hawkins states that, in the scirrhous tumor, the motion of the larynx is itself interfered with, and therefore it not only rises less freely, but respiration and deglutition of the patient are more affected than with most other tumors even when of much larger size. A large bronchocele may, however, seriously interfere with both these functions, so as even to cause death, but such extreme cases are very rare in this country. When the swelling extends laterally, these distressing symptoms are partly occasioned by the restraint of the sterno-cleido mastoideus muscle.

### VI. Alarming Syncope, from the Admission of Air into a Vein during an Amputation at the Shoulder Joint. By *Bransby B. Cooper*, F.R.S.

Eliz. Cousins, æt. 19, was admitted into Guy's Hospital for malignant disease of the right humerus. Mr. Cooper came to the conclusion that the only chance of saving life was by amputating the limb at the shoulder-joint. On the 23rd of May, the operation was performed by making a double flap, the subclavian artery being commanded by pressure upon the first rib: it occupied less than a minute; there was no loss of blood, and the patient bore it with great fortitude.

"The subclavian artery was immediately secured; but compression was still retained upon the first rib as there were small vessels requiring ligature. I then proceeded to remove a gland from the axilla, which was somewhat enlarged, and while dissecting it from its cellular attachments, I distinctly heard a peculiar gurgling noise, like air escaping with fluid from a narrow-necked bottle, and at the same instant the patient fell into a state of collapse, threatening immediate dissolution: the countenance was deadly pale, the pupils fixed, and inobedient to light; the pulse quite small and fluttering, although at intervals regular; the respiration hurried and feeble, and, at irregular intervals, attended with a deep sigh. The patient was directly placed in the horizontal posture, the flap covered over the wound, and retained by plaister. Cold water was dashed over her face, ammonia held to the nostrils, and a sponge filled with wine applied to the lips; but an hour elapsed before she was sufficiently recovered to be removed from the Operating Theatre.

"Upon being placed in bed, she passed her fæces and urine involuntarily;—some wine and camphor julep, with half a drachm of laudanum, were given to her. During the reaction coming on, she uttered a continual whining cry, and maintained a constant motion of alternate flexion and extension of the right leg, while the left remained perfectly quiescent. She continually complained of pain, extending up the right side of the head and neck. Her feet being cold, warm bottles were applied, and twenty drops of liq. opii sedativus given. At four o'clock the wound was dressed, when some small vessels were secured, and a nerve liberated which had been included in one of the ligatures; and to which, perhaps, the pain in the neck and head might be partly attributed. The edges of the wound were brought together and maintained in apposition by silk sutures and adhesive plaister. Twenty drops of laudanum were ordered: to be repeated if necessary.

"Wednesday, Eight o'clock, a.m.—When she awoke, all the symptoms were much relieved; pulse 150, small, irregular, and compressible. Tongue moist and white; profuse perspiration over the whole body—keeps her eyes constantly closed.

"Two, p.m.—The symptoms much the same as in the morning; still continual action in the right leg, with apparent loss of motion in the left. Pulse 140, feeble—seems inclined to dose, but answers perfectly coherently when spoken to—pupils still somewhat dilated—tongue moist—diaphoresis not so profuse. Beef-tea and arrowroot with wine ordered for her, when she desired it, as she takes nourishment freely.

"Eight, a.m., Thursday.—Has had a better night; dosing, but waking up at intervals with a whining cry—continues to keep her eyes constantly closed—diaphoresis less—pulse 140—still irregular and feeble—tongue moist—bowels have not been opened since the operation, but she voids her urine freely—action of the right leg continues; the left still motionless.

"On the 26th the stump was dressed—pus of an unhealthy character exuded from the wound, which, however, at the upper part had united for two inches.

"Her state was variable for the following six or seven days; the nights were restless, and there were occasional febrile exacerbations. The action of the right leg continued, and, on one occasion, there were involuntary flexions of the left, which she had not the power of extending.

"Opium was given freely with much relief, and she was allowed a nourishing diet.

"On June 3rd, she was much better, and from that time rapidly improved. Tonics and support of every kind were freely exhibited; the stump continued progressively to heal; and, on the 20th day after the operation, the ligature came away from the axillary artery.

"On the 25th day she was able to sit up in a chair and take her dinner. Complains of great numbness and loss of power in the left leg, which she drags after her; but it is as sensitive as the right, and while lying in bed, if pinched, she forcibly draws it up. On the 3rd of July, she was sufficiently recovered to leave the hospital, having no other unfavourable symptom than a slight dragging of the left leg." 46.

This patient was re-admitted into the hospital the latter end of November, 1843, for a tumor of the left scapula, of a similar morbid structure to that in the right arm. This tumor increased and destroyed life, by encroaching on the right side of the chest and on the spine, producing paraplegia. She died at the end of January, 1844.

This is a very satisfactory case of recovery from the alarming syncope consequent upon the accidental introduction of air into a large vein during an operation. The occurrence, though fortunately very rare, is one which the operating surgeon should be prepared to encounter, and to treat with energetic remedies. Mr. Cooper has appended to the above case a brief account of the results of the inquiries made by a Commission, instituted by the Royal Academy of Medicine, to investigate the circumstances of this accident. The author states that, the attention of surgeons was first invited to this subject by M. Beauchêne, but at what date he omits to mention, and adds in a note " it seems, however, that the fact of an injurious influence from the admission of air into the veins was known to Morgagni as long back as 1517, but had been forgotten, notwithstanding he has related a case." The date must be a misprint probably for 1715, since Morgagni wrote in the eighteenth century, not in the sixteenth. The statement is nevertheless interesting, but would have been more satisfactory if a reference had been given to the authority upon which it is made.

### VII. Account of a Horn developed from the Human Skin; with Observations on the Pathology of certain Disorders of the Sebaceous Glands. By *Erasmus Wilson*, Esq.

Mr. Wilson commences this paper by a description of the mode of production of the sebaceous secretion and its microscopic appearances. He remarks :—

" The sebaceous substance is secreted from the blood, through the agency of the cells which compose the epithelial lining of the gland, as is the case probably with all the secretions of the body; but there is this difference between the sebaceous and other secretions, namely, that the former is semi-solid, while the rest are fluid; the solidity or density of the sebaceous matter being due to the great number of empty and more or less distended cells which compose its mass." 53.

The quantity of this matter varies in different individuals as to its density and apparent composition, and its cells undergo changes in accordance with the state of health of the skin, or of the individual, and perhaps also in conformity with the chemical constitution of the blood. Mr. Wilson alludes to two of these changes, one which occurs in molluscum contagiosum, a disease consisting in the development upon the skin of small sebaceous tumors in variable numbers. In this affection the tumor results from the solidity of the contents of the cells of the sebaceous secretion, the solidity being so great as to preserve the form of the distended cells, and consequently to dilate the follicle with the ducts of the sebaceous glands. There is besides, a deficiency of oil globules and of albuminous fluid, and consequently the impacted substance is dense and dry. The contents of the cells in this disease are chiefly coagulated albumen in a granular form. The second modification in the constituents of the sebaceous cells is that which was described by Mr. Dalrymple in the last volume of the Society's Transactions. In this case the sebaceous cells were flattened, having the ordinary appearance of epithelial scales, and containing phosphate and carbonate of lime in their interior. The author next alludes to another pathological state consequent on imperfect secretion of the sebaceous substance, in which, from the torpid action of the skin, or from the nature of the contents of the cells, or from both causes acting together, the sebaceous substance collects within the follicle, becomes impacted, and acquires an abnormal degree of density.

"In this situation the impacted mass exerts so great an amount of pressure on the vascular walls of the follicle, as to abrogate its special function, and the peculiar elements of the sebaceous secretion cease to be produced. The formation of epithelium, however, still continues, and layer after layer of epithelial scales are developed, until the mass acquires considerable size. Tumors of this kind, from the nature of the position of the sebaceous follicle, namely, within the corium, rarely acquire a large size as compared with tumors in other situations. They are prevented from pressing inwards by the deep stratum of the corium; the same structure opposes their increase outwardly or laterally. Nevertheless, I have seen a tumor of this kind which measured three-quarters of an inch in diameter, but not more than a quarter of an inch in thickness. The aperture of the follicle remains open, and is more or less distended in proportion to the extent of the tumor; but, from the nature of the collection, there is no tendency to its escape. I have called such tumors *sebaceous accumulations.* Certain minute tumors, commonly met with in clusters around and upon the eyelids, *sebaceous miliary tubercles,* are of the same pathological nature with the sebaceous accumulations, but in these the excretory follicle is closed.

"The peculiar pathological character of the tumors just described is their laminated texture, and the identity of structure of their contents with epidermis, most, if not all, of the peculiar constituents of sebaceous substance being absent."

"If now, in the cases above recited, we imagine the upper wall of the laminated tumor to be removed, and the accumulated substance exposed to the influence of the atmosphere, any moisture retained by the epithelial laminæ would soon become dissipated, and the whole mass would acquire the consistence and hardness of epidermis of equal thickness; in other words, it would be converted into horn.

"Such a case as that which I am now supposing does sometimes in reality occur. The aperture of the follicle acquires an unusual degree of dilatation, and some of the hardened contents of the tumor are pressed through the opening. By the addition of fresh layers from below, (the formative power having increased by the removal of superficial pressure,) the indurated mass is still further forced outwards, dilating the aperture as with a wedge, and finally increasing its size to that of the entire base of the hypertrophied follicle. The process of formation of new epithelial layers by the walls of the follicle (now become the base of the mass) will go on, unless interrupted by surgical means, for years, and in this manner those singular bodies, of which so many remarkable examples are on record, *horns,* are produced." 59.

Mr. Wilson relates a well-marked example of horn in a female servant fifty-seven years of age. At the age of five-and-twenty, "she observed a small elevation, like a pimple, on the site of the present growth; the pimple increased in size, was somewhat painful, and in about ten years from its first appearance burst, and discharged a quantity of matter resembling 'mashed potatoe.' From this moment a cavity always remained, from the bottom of which some 'scurfy' matter could be raised by the finger nail. At the beginning of the current year the present growth made its appearance in the situation of the cavity, and increasing in size, gave her much pain and uneasiness. The skin around it was red and inflamed, and she applied a poultice, which had the effect, according to her, of making it grow still faster. During the summer she suffered much from the frequent jerks which the growth received from her dress, and from awkward blows which it sustained, and in the month of October she applied to her master for relief. At this period the growth had acquired a considerable size: it was situated on the upper and front part of the thigh, and presented the appearance and characters of horn. It was semi-transparent, yellowish in colour, dense and horny in texture, ribbed on the surface, insensible to the pressure of the nail, and firmly rooted in the skin. In general appearance it resembled the broad and curved beak of a bird, of large size, and had a broad and extensive base. Around the base, the integument arose to the height of several lines, and in two places to fully half-an-inch. The skin was thin and attenuated as though from the effects of stretching, the epidermis being continuous with the surface of the horn, and gave the idea of a degeneration of the integument into the horny structure."

Mr. Wilson removed the horn by cutting through the integument round its base, and dissecting it from the subcutaneous tissue. The sore healed slowly, but was closed by the fifth week. On examining the horn after removal, the author "found its base to be formed by the deep stratum of the corium, so that it was obviously a cutaneous formation. The base was oval in shape, and measured in its long diameter one inch and a half, and in the opposite direction one inch and a quarter. The horn was two inches and three-quarters in length, by two inches in greatest breadth, and its elevation above the surface was one inch and a quarter. The latter measurement was that of the vertical thickness of the horn; for in consequence of its mode of growth its long diameter lay parallel with the surface of the skin. The sebaceous accumulation must originally have formed a prominent tumor, from the side of which the protrusion took place; the thin integument covering the other half still retaining its elevation from distension. Traces of this mode of formation are still apparent upon the surface of the horn. Subsequently, the thin integument has become inflamed and ulcerated, and, receiving no granulations from beneath, has desiccated upon its horny contents. This ulceration was the cause of the redness and pain of which the patient complained, and its extent is marked upon the horn, by a rough discoloured surface of a circular figure, surrounded for more than two-thirds of its extent by a margin of thinned integument. The weight of the horn was six drachms.

"The section of the growth presents all the characters of horn; it is laminated longitudinally, the laminæ being distinctly traced by their difference of tint from the base to the apex of the horn. At the apex, moreover, it is split in the direction of its laminæ, and several external lamellæ are partly separated from those beneath."

This description is followed by an account of the minute structure and size of the epithelial cells of which the horn is composed, to which we must refer those of our readers who are interested in the subject. Horny growths from the skin do not appear to be so uncommon as is generally believed. Mr. Wilson has collected ninety cases, of which forty-four were females, and thirty-nine males; of the remainder the sex is not mentioned. Forty-eight were seated on the head, four on the face, four on the nose, eleven on the thigh, three on the leg and foot, six on the back, five on the glans penis, and nine on the trunk of the body. Old age appears to be a predisposing cause of this affection, as is shown by the greater frequency of its occurrence in elderly persons. The author refers to some remarkable cases of human horn on record. Amongst others, he mentions the case of a Mexican porter, who had a horn upon the upper and lateral part of the head, which was fourteen inches in circumference around its shaft, and it divided above this point into three branches. Sir E. Home saw two cases, in both of which the growth measured five inches by one inch in diameter. They were curled, and had the appearance of isinglass. In one case the horn was fourteen years growing. A horn in the British Museum is said to measure eleven inches in length by two and a half in circumference. The paper is concluded by a reference to the principal writers who have paid attention to the subject of these growths.

Mr. Wilson is known to have devoted a good deal of attention to the investigation of the diseases of the skin, and it is only justice to him to add, that in the observations which we have just brought under the notice of our readers, the development of horn from the skin is satisfactorily explained, and its structure ably described, and that the paper affords evidence of considerable research.

### VIII. On the Early Organization of Coagula and mixed Fibrinous Effusions, under certain Conditions of the System. By *John Dalrymple*, Esq.

In the 23rd volume of the Society's Transactions, Mr. Dalrymple has described the case of a seaman who died of scurvy on board the Dreadnought hospital ship. One of the legs of this man having been injected by Mr. Busk, it was found on examination that a large coagulum of blood extravasated beneath the periosteum of the tibia was minutely injected. This specimen was adduced in order to shew the rapidity of the organization of the fibrinous materials of the blood, in certain cachectic conditions of the system. In Mr. Travers' recent work on inflammation, that gentleman expresses a doubt as to the character of the effusion in the case referred to by Mr. Dalrymple, and inclines to the belief, that the injected mass was rather a fibrinous effusion mixed with the colouring principle of the blood, than a true extravasation, and that the injected canals were the original vessels between the periosteum and the bone, stretched and separated by the effused fibrin. It appears that Mr. Busk also conceived that the specimen consisted, in part at least, of effused fibrin, but he maintains the new formation of the injected vessels. The object of the present paper is to confirm the fact stated in the former one, and to meet the objections of Mr. Travers by further and more satisfactory observations. A Lascar died of scurvy on board the Dreadnought, in whose knee-joint were found many coagula, some adherent to the reflected synovial membrane surrounding the cartilages of the femur and tibia, and some loose in the cavity of the joint. The limb having been very successfully injected, the attached coagula were found to be permeated with new and numerous capillary vessels. In the former case, the fact of the organization rested solely upon the perfection of the injection, the absence of any extravasation of the vermilion, and the form and peculiarity of type of the new vessels. In this more recent case, Mr. Dalrymple, instead of relying solely upon the fact of the clot having been injected, proceeded to a microscopical examination of the morbid parts.

" The coagula presented the appearance of dark but firm clots, and upon being viewed beneath the microscope, their colour was found to depend upon an infinity of red blood disks in an entire state, mingled with fibrinous globules. The firmness of the masses, however, was due to the advancing organisation of the fibrin itself, the fibrinous cells being found in all stages, from the granulated sphere to the caudated cell, ultimately developing into filamentous tissue.

" There were—

" 1. The exudation or fibrinous corpuscules, spheroidal and granular.

" 2. Nucleated cells—oval with excentric nuclei and nucleoli.

" 3. Cells elongating in one direction and becoming caudate.

" 4. Cells more elongated, and the tails occasionally bifid.

" 5. Cells drawn out into a filamentous prolongation at either end. And,

" Finally, their conversion into simple wavy filaments.

" At first the cells were filled with granular matter, as well as with their large nuclei; but as they increased and became elongated, the nuclei diminished, and the granular matter was less abundant: at length the nuclei nearly disappeared, and the filaments became clear. It should be added, that all these varieties of cell-development were seen in one and the same preparation, and at the same time.

" It was to the interlacement of these caudate cells and filaments, that the firmness and definite outline of the coagula were due; and this description exhibits the true progressive organisation of the living germs, which *precedes*, as I believe, the formation of new vessels.

" In corroboration of this last remark, I may observe the curious fact, that in the loose coagula found in this same joint, there were not only present those appearances, described by Mr. Gulliver, as due to the simple coagulation of blood out of the vessels, viz. its fibrilation intermixed with blood disks and fibrinous globules, but a distinct stage in advance, or an attempt at progressive organisation, although the coagula were loose in the joint, and unattached to any living tissue.

" Even here, a few caudate cells were found, intermingled with coagulated and fibrilated blood, enough, however, to show that the law of vitality impressed upon the cell germs was in action, after all direct connection with living tissue had ceased, and when it is obvious no new vessel could have been formed within the mass. This is a point that requires extended observation, and may have some connection with the obscure subject of the production of loose cartilages, sometimes found in joints and bursal cavities." 73.

Pathologists accustomed to microscopical inquiries, after reading this description, will entertain little question but that the fibrin submitted to examination was in a state of progressive organization, and if, as there appears no reason to doubt, the coagula were really effused blood, Mr. Dalrymple may be said to have established the important fact which he formerly affirmed. It is not, however, now contended, nor was it in his former paper, *that ordinary extravasations of blood in the healthy body* become organized, because, he observes, the vitality of surrounding parts is higher than that of the blood so effused, and the absorbents, under such conditions, effect their ordinary changes in consequence of a tendency to disintegration, rather than to an advanced development of the effused blood.

### IX. CASE OF EXTIRPATION OF AN OVARIAN CYST TERMINATING FATALLY. By *Bransby B. Cooper*, F.R.S.

As full details of several cases, in which enlarged ovarian cysts have been extirpated, have been published within the last few years, it will be sufficient to give a brief account of this case, and of a similar one which forms the subject of the next paper, and the remarks which they naturally suggest may be reserved till we come to consider the observations of Mr. Phillips on the recorded cases of this operation. The patient was a healthy-looking woman, æt. 32, who, though married, had never been pregnant. An abdominal enlargement was first noticed about five years ago, but she could not discover on which side it commenced. Eleven months afterwards she was admitted into Guy's Hospital, " to have the tumor extracted, which, however, was not performed, as the operation was unsuccessful in a case at that time in the hospital She consequently went out, and a month afterwards married, but at the expiration of six months her general health became so much impaired from the rapid accumulation of fluid, that she applied to me to perform paracentesis abdominis; but a day or two before her admission into the hospital for the purpose, having taken, by the advice of a friend, a glass of gin, she passed a gallon of urine during the night, and the increased flow continued for ten days afterwards, although in diminished quantities; so that at the end of that period no vestige could be discovered of the original tumor. Many months, however, had not elapsed before her abdomen again enlarged, and continued to do so for eighteen months, when she again passed a large quantity of urine, and the swelling was a second time dispersed, but not so completely as on the former occasion. Twenty months after this last favourable event, the tumor having acquired its original bulk, paracentesis abdominis was performed, and three gallons of a straw-coloured fluid were drawn off. She recovered from this operation without a bad symptom. Seven months ago she was again tapped, an interval of thirteen months having elapsed between the two operations. On this occasion she likewise convalesced without anything untoward having occurred; but the fluid was of a darker colour, and thicker. It does not appear that she has ever had symptoms which would induce the belief that she had suffered from peritonitis, so as to cause any adhesions."

The abdomen to the right, is stated to have been larger than at the full period of utero-gestation, and of an oval form, and on passing the hand over it, an irregular mass, of the size of a saucer, was felt midway between the ensiform cartilage and umbilicus, composed probably of compound cells. Over every part of the abdomen there was dulness on percussion, and more than usually distinct fluctuation.

The state of the general health appeared to be good, and Dr. Blundell, who saw the patient, gave it as his opinion that the case was a favorable one for the removal of the tumor. The operation was therefore decided upon.

The temperature of the room having been raised to F. 72°, and the patient placed in a convenient position, "an incision was made below the umbilicus, in the median line, between three and four inches in length; and the peritoneal cavity being opened to a small extent, a little ascitic fluid escaped. The finger was introduced, and passed around the opening, and a few slight adhesions broken down. An incision was now made through the integuments, commencing three inches below the ensiform cartilage, and extending to the upper part of the first incision, carefully avoiding the umbilicus; and the sub-cutaneous structures were then divided by a probe-pointed bistoury, cutting from below upwards, the finger being used as a director. The wound was also enlarged towards the pubis. Very trifling hæmorrhage ensued; and the tumor, now clearly exposed, slowly and steadily advanced, its substance being supported very carefully by Mr. Law, whilst Dr. Noyes kept the integuments in close contact with it posteriorly, in order to avoid unnecessary exposure of the intestines, or other viscera. A broad and thin pedicle, connected with the right ovary, was now fairly brought into view, and a strong needle fixed in a handle, and armed with double silk, was passed through its centre, and both ligatures being securely tied, it was divided between the ligature and cyst, and the mass removed. Dr. Cape having examined the remaining ovary, and pronounced it healthy, the wound was closed by about fifteen interrupted sutures; and strips of adhesive plaister being applied, and the roller adjusted, she was removed to bed.

"The patient bore the operation with uncommon fortitude, but there being some slight attempt to vomit during the application of the sutures, half a drachm of Battley's solution, in camphor-water, was given. Pulse before the operation, 87; after removal to bed, 96."

She appeared to be going on well till the second day after the operation, when symptoms of peritonitis occurred. It assumed an asthenic character, and terminated fatally on the seventh day. The cyst is stated to have been a collection of compound cells, which weighed 32lbs., but the description given of it is very imperfect. On examination of the body, the intestines were found coated and glued together with lymph, and in the interspaces formed by the adhesions there was a small quantity of purulent fluid. About an ounce of blood was observed in the right iliac fossa, probably effused from the divided vessels of the peduncle. The uterus was large, tumid, and of a dark colour; anteriorly, and at its superior part, there was a soft fungoid tubercle, of the size of a walnut. This was examined under the microscope by Mr. Reynolds, and pronounced to be highly malignant. A small piece of omentum, adherent to the peduncle, was enclosed in the ligature.

The age and health of the patient, and condition of the tumor, were favorable for the operation. The only untoward circumstance was the disease in the uterus, which could not have been ascertained beforehand.

X. CASE OF THE REMOVAL OF A DISEASED OVARIUM, TERMINATING FATALLY ON THE SEVENTH DAY AFTER THE OPERATION. By *T. M. Greenhow*, Esq.

Mary Nicholson, æt. 29, had enjoyed good health till her marriage two years ago, except that, for about two years previously, she had frequent uterine discharge of blood. Shortly after marriage the discharge returned, and about six months afterwards she first felt a swelling in the pubic region, which extended to the right side, and in a short time a moveable tumor, about the size of an orange, could be distinguished. The swelling from this time rapidly enlarged, and was attended with almost constant uterine discharge, under which her strength gave way. She had once been tapped, but little else than blood in moderate quantity was removed, and, since the operation, no uterine discharge has taken place. At present her strength is much increased, and she has no symptoms of constitutional disorder. "The abdomen is about as large as at the full period of utero-gestation; fluctuation in one or two situations; but generally the tumor is firm, and feels as if divided into two separate masses. She has no pain or tenderness in any part of abdomen, except at one point towards the right iliac region, where the original moveable swelling was first felt. No alteration in the os, or cervix uteri can be discovered, and, as far as can be ascertained by examination *per vaginam*, the organ is in a natural condition."

The operation was performed on September 3rd, at 11, A. M. The temperature of the room having been raised to 76°.

"The incision reached from a little below the ensiform cartilage to near the pubis. The peritoneum was opened a little below the umbilicus, near the scar left by the trocar when she was tapped, in the expectation that at this part some adhesions would probably exist. This was the case, but they were easily separated. The incision was completed upwards and downwards with a bent bistoury through the peritoneum, directed by two fingers. Several adhesions existed in different parts of the tumor, which now became fully exposed. The principal one however was to the omentum, which was spread over the upper part of the right side of the tumor, and was closely united to it. This adhesion was divided with the bistoury, and then the tumor, with some effort from its great size and weight, was raised from its situation. Being carefully supported by Mr. Frost, while Mr. Heath closed the wound as it passed out, and retained the intestines in their place, I was enabled to pass double ligatures through the pedicle, and having firmly tied them, divided it close to the tumor, which was thus liberated from its attachments, and removed. Two arteries bled freely, one in the divided omentum and the other in the pedicle; these being carefully secured, the wound was brought together by sutures and adhesive plaister, compresses of lint and linen were placed over the abdomen, and a many-tailed bandage, which had been placed under the patient in readiness, secured the whole. The operation was well borne by the patient, though she vomited towards the end several times. This however she attributed to some spirits of ammonia, and brandy-and-water, occasionally given to obviate faintness, which she says always made her vomit. The pulse remained firm, and within half an hour from being placed on the table she was again laid in bed. The quantity of blood lost did not exceed ℥vj. After being put to bed she complained much of pain and smarting of the abdomen. Mur. morph. gr. ss. in camphor mixture was given, which induced a tendency to sleep. The pulse varied from 72 to 90. After half an hour, the pain continuing severe, the opiate was repeated. The strictest injunctions as to quietude and diet were given, the latter being confined to barley and toast water." 92.

We have not space for the minute account of the subsequent progress of the case given by Mr. Greenhow. Symptoms of inflammation in the abdomen arose. She gradually got worse, and died at 2, A. M., on the 9th, the sixth day after the operation. On examination of the body, the wound was found nearly healed throughout. On laying open the abdomen, the omentum presented itself adhering to the intestines and its folds matted together. General, but not very strong, adhesions existed between the folds of intestine as well as to the walls of the abdomen. In addition to these, and other marks of peritonitis, the pylorus exhibited a distinct inflammatory blush, which extended two or three inches into the duodenum, gradually diminishing in intensity. In this situation, the mucous membrane was softened, and exhibited numerous points of ulceration. Two or three patches of similar vascularity and softening showed themselves at long intervals in the course of the small intestines, but without any ulceration. The peduncle to which the tumor had been attached, proved to be the left broad ligament of the uterus, and it appeared that the diseased ovarium, after attaining a certain size, had passed over to the opposite iliac region, the uterus at the same time making a semi-turn on its own axis, so as to place its dorsum in the anterior position. A small portion of coagulated blood was found in the pubic region. The right ovarium and the uterus were healthy, but the cavity of the latter was lined with a fine vascular membrane resembling the decidua. The tumor, with the exception of one or two small cysts containing a few ounces of yellow fluid, was firm and solid and the size of the uterus before delivery. It weighed 12lbs. 7 oz. avoirdupois.

"The general structure is cellular, but in many parts it is very dense; a number of small cells or cysts, however, pervade its substance, besides the larger ones already mentioned. One of these cells, about the size of a walnut, near the centre, contained a brownish pulpy substance, resembling thickened pus. The centre of the mass, for the space of several inches, was of a bright red colour, as if an active circulation had been carried on in the interior. The largest cyst, at the upper part of the tumor, was white and glistening in its interior, resembling the ordinary character of ovarian cysts; but the dense solidity of the general mass, and vascularity of the interior, distinguish this tumor from the other instances of ovarian disease which have come under my notice." 100.

Owing to the reduced condition of the patient from discharges of blood, this case was not so favorable for an operation as the preceding one. Mr. Greenhow believes that her convalescence after the inflammatory attack was very nearly established, and that, but for the diseased condition of the pyloric extremity of the stomach, her recovery would have been effected. At so early a period as on the sixth day* after the operation, and with unabsorbed lymph and coagula of blood in the abdomen, convalescence cannot be said to be established, and we must regard the morbid state of the pylorus as one of the results of the operation, probably induced by, and

* In the heading of the case, the day of death is erroneously stated to be the seventh.

not the cause of, the severe vomiting from which the patient suffered. Mr. Cooper and Mr. Greenhow are entitled to the thanks of the profession for fairly and faithfully recording the particulars of these unsuccessful cases. It is right to add that, in both instances, the operation appears to have been performed with every care and precaution.

The following paper, though the last in the volume, may be advantageously considered after these two cases.

XI. OBSERVATIONS ON THE RECORDED CASES OF OPERATIONS FOR THE EXTRACTION OF OVARIAN TUMORS. By *Benjamin Phillips*, F.R.S.

In this paper, the author has brought before the Society all the information which he has been able to collect on the subject of the extraction of ovarian tumors, in order to afford some aid in coming to a right judgment, whether this operation shall be classed amongst the benefits conferred by science upon man. It is remarked, that the opinion of any man, however eminent, pronounced without a careful estimate of the materials which have now become available to us, cannot and ought not to determine the question. He observes, that it has been the fate of some grave and dangerous operations, to be received into favour and admitted into practice from the moment they were proposed; and of others, less grave and dangerous, to be assailed by great prejudice; whilst those who have desired to afford them a fair trial, have been exposed to censure and contumely; in either case the results of the operation being disregarded.

"In 1818, Mott placed a ligature around the arteria innominata, and the operation was unsuccessful; and yet, formidable as is the operation, and unvaried the failures in every instance, though performed by the ablest surgeons, it has been repeated twelve times, and still I have no doubt that men of the greatest eminence and most unquestionable skill would nevertheless resort to an operation the almost certain result of which would be death. The extraction of an ovarian tumor is much more simple, much less grave, than the ligature of the innominata, and yet by a large portion of the profession, and by some of the most celebrated of its members, it has been denounced as rash and imprudent. With a few exceptions it has not been performed by the more experienced and able surgeons either in this country or on the Continent; it has fallen into the hands of men less accustomed to perform great operations, and it may be not unreasonably assumed less conversant with the after-treatment which may be needful. Although performed many times in London, I believe it has been introduced into no other Hospital than Guy's, and even were its performance more frequently called for, and its success more decided, it would hardly be classed amongst the recognised operations of surgery until warranted by the experience of Hospital practice." 471.

The operation of tying the arteria innominata for the cure of aneurism cannot be fairly compared with the operation for the removal of an ovarian tumor. In the first case the disease is so dangerous, that death is almost certain within a brief period, and the surgeon knowing from his experience of the efficacy of ligatures on arteries in cases of aneurism generally, that the operation affords the only chance of saving life, offers it as a *dernier ressort* to his patient. The next case may be a successful one, and if, in one case in thirteen, or even one in twenty, life is preserved, the operation is warranted; and it is so upon precisely the same principle, that in the case of two limbs irretrievably damaged by violence, we should amputate if necessary both thighs or both arms as the only chance of rescuing the sufferer from death—poor as that chance may be. But how different are the circumstances of a person with an ovarian tumor. The danger is distant—the patient will probably live at least four years, and she may survive twenty or even longer, whereas, if an operation be performed, the chances are nearly even that she dies in a week. We have a patient who has a large abdominal tumor which has existed for years, and been stationary for the last two. She passes her days in comparative ease, is able to perform the common offices of life, to enjoy society, and even to tend a sick husband. Until this can be said of a patient with a subclavian aneurism, no fair comparison can be drawn between the circumstances of these two cases.

Mr. Phillips gives a Table of eighty-one cases, in which are exhibited, the names of the operators, the ages of the patients, the nature of the operations, the state of the tumors, the results, and remarks on the cases. The Table includes cases operated on in this country as well as on the Continent. It would have been more complete and useful, if the authorities had been quoted. In sixty-one cases, the tumor was extracted: in fifteen cases, adhesions, or other circumstances, prevented its removal; in five instances no tumor was found. Of the cases in which the operation was completed, the tumor being extracted, thirty-five terminated favourably; the patient recovered: in twenty-six instances the termination was unfavourable; the patient died. Of the five cases in which no tumor was discovered, all recovered. Of the fifteen cases in which adhesions, or other circumstances, prevented the extraction of the tumor, nine recovered, six died. Mr. Phillips very justly observes, the proper way "of looking at this plan of treatment, is to observe the number of cases submitted to operation, and the number of recoveries after the removal of the tumor. I conceive this to be the fair way, because, what has happened already is, in my judgment, likely to happen again. Adhesions may be too strong and extensive to make removal prudent; the tumor may be other than ovarian, or it may be that no tumor can be found. Regarded in this light, it appears that the operation has been performed eighty-one times, and that in thirty-five instances the patient has recovered after the extirpation of the tumor. It is true, that forty-nine patients survived gastrotomy; but many of them were subjected to such a painful and dangerous operation, on the one hand without necessity, and on the other without being disembarrassed of the disease."

In order to estimate the relative advantages and dangers of the major and minor operations, Mr. Phillips has made two Tables, one including the cases in which the tumor was removed entire, the incision having an extent of six inches or upwards: the other, including those cases in which part or the whole of the fluid was evacuated before the extraction was attempted, the length of the incision having been under six inches. Of the former, there are fifty-five cases, twenty-three of which were successful: of the latter, there are twenty-seven instances, of which thirteen were successful.

In the consideration of these cases, the following points arise.

"First.—Can we determine, with certainty, whether a tumor be ovarian or not? If not, have the failures been so frequent as to constitute a reason why the operation should not be attempted?

"Second.—Supposing a tumor to exist, and to be ovarian, can we ascertain the nature of its contents, as well as its connections? If not, have the failures been so many as to be an objection to the adoption of the operation at all?

"Third.—Are the results of this plan of treatment sufficiently favourable to justify us in preferring extirpation to any other mode of treating ovarian tumors? And if so, what plan of operation promises most success?" 481.

In proof of the uncertainty of the signs of ovarian tumor is adduced the fact, that out of the eighty-one instances in which the operation for extirpation was attempted, five times, at least, the abdomen has been laid open, and no tumor discovered—and in six others the tumor was not ovarian. The patients recovered in all the instances in which no tumor was found, and hysterical or other tympanitis, or tumors of the omentum were the sources of error in the cases referred to. Though it must be acknowledged that in many instances the diagnosis of abdominal tumors is extremely difficult, yet many cases occur in which the surgeon can positively predicate the existence of ovarian disease, and we need scarcely say that, it is only in such cases where the nature of the tumor is plain and indisputable that an operation should be thought of.

With regard to the second point for inquiry, Mr. Phillips says "we have no certain means of ascertaining the contents and connections of tumors presumed to be ovarian. In six instances, the abdomen has been laid open, and the tumor has been found to be either diseased omentum, or diseased or gravid uterus. Many times a presumed ovarian cyst has been punctured, and no fluid has escaped; the operation has been a 'dry tapping.' I have been present when an operation has been performed for the extraction of the cyst, where, although the examination was made by men of tried ability, who had no doubt of the contents being fluid, yet not a drop of fluid was contained in it—it was a stiff jelly.

"With reference to connections or adhesions, the difficulties met with are still more formidable. In fifteen cases where the operation for extraction was commenced, it was found necessary to discontinue it, in consequence of the extent of the adhesions. In twenty-five other instances adhesions existed. Of the fifteen cases in which they caused the abandonment of the operation, six terminated fatally."

A Table is given of these fifteen cases of "*no extraction*." Upon the subject of adhesions we find the following just remarks.

"This seems to me to be the pinching point of the case. I would admit, that a very careful and competent observer would not be likely to fail often, in his conclusions as to the existence or non-existence of a tumor, and as to its being ovarian or not; I would admit that he would not often fail in the determination of the contents, but I know no sure means of ascertaining whether there be extensive adhesions or not: in most cases, there are no physical signs by which we can determine this. Is it not certain that we can, in ordinary cases, cause the abdominal parietes to glide over the surface of the tumor; in many cases, persons are deceived by the gliding of the superficial upon the deeper-seated layer of the abdominal walls, and if we could, it would only avail for its anterior surface. The crepitating sign pointed out by Dr. Bright is only present when the adhesions are recent; and as to the motion of the tumor with the diaphragm, considerable adhesions may exist without much interfering with it. An examination per vaginam would not set the question at rest. Our main reliance is therefore upon the signs of peritonitis—if the evidence be clear that peritoneal inflammation has existed, it is probable that adhesions are present; but we may find adhesions where there has been no reason to suspect peritonitis. Still extensive adhesions in the absence of symptoms of peritonitis are by no means common. It is then mainly upon this point that we must rely before proceeding to operation." 485.

We quite agree with the author that the adhesions are rarely extensive without previous symptoms of peritonitis, and, allowing the extirpation of the diseased ovary to be a justifiable operation, we think that, in the absence of these symptoms, it may be undertaken with every prospect of being completed, and the operator may find encouragement in the fact furnished by Mr. Phillips's tables, that of the forty cases in which adhesions were found, twenty-six survived, which is at the rate of sixty per cent., whereas of the whole eighty-one operations, forty-seven recovered, or fifty-eight per cent. The great danger of this operation arises from peritonitis, and so far as our present experience warrants a conclusion, that danger is not increased by previous inflammation of the serous membrane and its consequences, adhesions, though their existence necessarily leads to greater disturbance of the important parts exposed in the operation.

The next point considered is whether the results on record justify us in preferring extraction to any other plan of treatment. We know that the operation for extraction is one of a very serious nature, but have we any other means of cure to propose? All internal and external remedies have failed. We may advise the patient to bear her sufferings, reminding her that large tumors of the kind have remained stationary for twenty years and more. But these are exceptions, and when the case gets worse, will you advise tapping, or the more serious operation of extracting the cyst? The tumor may not be fluid, but if it be so and you tap it, the cyst becomes refilled, it may be slowly, but usually in a few weeks, and thus repeated tapping is necessary, the intervals between the operations diminishing until the patient dies, worn out, the average duration of life from the first tapping not exceeding four years. On the other hand, "extraction, though not a very painful, is a dangerous operation: the experience we possess justifies us in the expectation that, in forty-four cases out of one hundred, the tumor may be extracted, and life saved; but at the same time it cannot be concealed that, out of the eighty-one operations to which we have referred, thirty-two died, and that soon—in fact in a few days."

The author remarks that, in many cases where disease affects one ovary to a large extent, it affects the other, though to a smaller extent, and it is possible that when the disease is removed from one ovary, the nutritive action which was previously directed upon it, may afterwards be concentrated on the remaining point, and cause its rapid development. This ground of apprehension appears to us chimerical. It would be as reasonable to decline an operation for lithotomy because of the liability which must remain to the formation of a fresh concretion, as to object to the extirpation of one enlarged ovary, in consequence of the remaining one being liable to become affected with a similar disease.

There is one material point bearing on this operation, overlooked by Mr. Phillips, which it may be as well to notice here, viz. whether the cystic disease to which the ovary is subject, is of a malignant character or not. Mr. Cooper states, in the paper which we have reviewed, that Dr. Bright, Dr. Hodgkin and Mr. T. King believe it to be so. There can be no doubt but that the ovary is liable to malignant disease, and that a malignant growth sometimes co-exists with the cystic disease; but if we could come to the conclusion that ovarian cystic tumors are generally of a malignant nature, which is known to be the opinion entertained by Dr. Hodgkin, we should at once decide against the propriety of extirpation in any case, for it would always be impossible to ascertain beforehand that the disease is limited to the ovary. But, notwithstanding the high authority of Dr. Bright and Dr. Hodgkin, we have no hesitation in expressing the opinion that the ordinary cystic enlargement of the ovary is not of a malignant character, and may be extirpated without risk of leaving behind the germs of future disease in other parts.

With regard to the operation which should be selected, a point on which it appears that much difference of opinion exists, the calculations from the table tell, as we have already shown, rather in favour of the minor operation; the proportion of recoveries being forty-eight per cent, whilst in the cases of the major operation it was forty-two. Mr. Phillips's observation of the smaller incision is much more favourable: the operations being six, the successful cases five. Cases, however, may occur, in which an enlargement of the incision is absolutely required, as when the contents of the cyst, instead of being fluid, prove to be solid; but he observes, that circumstance does not in any way militate against the plan of making as small an incision as is consistent with the easy removal of an emptied cyst, provided it be large enough for the convenient application of the ligature round the peduncle. The statistics of the operation at present are by no means sufficient to decide this question. The length of the incision seems to us of less importance than the gentle manipulation of the parts, as we believe that the risk of peritonitis is very slightly if at all increased by the extension of the incision, certainly not so much so as by any force or rough handling which may be necessary if the space allowed for the manœuvres of the operator be too limited.

The author's tables are defective in some particulars, which it is impossible for him at the present time to supply, but which we hope may be obtained at some future period, as they are of the utmost importance in determining the utility and necessity of the operation. It is desirable to know the duration of life and state of health enjoyed by patients after the operation, and whether persons, one of whose ovaries has been extirpated, have suffered any serious inconvenience from the incision in the abdomen and adhesions between the viscera, the result of peritonitis.

It must be observed, in conclusion, that the members of the Society, and indeed the profession, are under obligations to Mr. Phillips for the information which he has submitted to their consideration, and the importance of the subject and the many interesting points *sub judice*, upon which the facts accumulated in this paper bear, have led us to quote largely from it. It is not difficult to perceive that the tenour of the author's remarks is in favour of the operation, but it might have been expected that he would have given an opinion on the subject, based on the facts which he has taken so much pains to collect. Mr. Phillips has himself extirpated an ovarian cyst, has witnessed its performance by others in several cases; he is a lecturer on surgery, and is known to have paid considerable attention to the statistics of operations. We think therefore that he might without presumption have expressed something like a confident opinion on the propriety and value of the operation. For our own part we have for some time come to the conclusion, which is rather confirmed by the facts here presented to us, that, under the circumstances of a disease which, on the one hand, may not destroy life for years, and commonly admits of palliative treatment and temporary relief; and, on the other, can only be radically cured by means which it is nearly an even chance will destroy life within a week, the surgeon is not warranted in recommending so dangerous an operation to his patient. At the same time we do not altogether object to its performance. It sometimes happens that persons have to decide for themselves on what terms they will continue to enjoy life. What is tolerable to one may be intolerable to another, and many look with dismay on an operation to which others would cheerfully submit in order to be relieved from distress and inconvenience. A person with a large incurable ulcer on the leg, or with a permanent contraction of the knee-joint, will sometimes desire amputation rather than bear the constant annoyance of these complaints, and though the operation is one dangerous to life, the surgeon is held to be justified in complying with the wishes of the patient in performing it. The case of an ovarian tumor appears to us to belong to this class. We should not recommend the operation, but if a patient of sound constitution be anxious, after the risks of an operation have been fairly stated to her, to incur these risks in order to get rid of so serious, and in the end so fatal a malady as this disease, it is the duty of the surgeon to operate. We know of a case of recent occurrence in the country, in which a talented surgeon most reluctantly undertook the operation at the urgent desire of the patient. The result was perfectly successful, and no one who contrasts the present healthy condition of this person with her former state and prospects, notwithstanding the dangers incurred, can question the discretion of the operator in yielding to the pressing wishes of the sufferer.

### XII. On the State in which the Uric Acid exists in the Urine. By *Henry Bence Jones*, M.A. Cantab.

The author first gives the opinions of the chief chemists on this subject. Berzelius mentions Dr. Prout's view of the uric acid existing as urate of ammonia, and then states his own, that uric acid most frequently is in an uncombined state; but perhaps modified by the presence of other matters. Dr. Simon thinks that urine may contain free uric acid, and also urate of ammonia. Becquerel says, that the ordinary fine amorphous powder which is deposited from acid urine, consists of uric acid combined with colouring matter and the (so called) extractive matters of the urine. We shall not follow the author in the analyses detailed in this paper, but must refer those interested in the subject to the work itself. It will be sufficient to observe that, from his experiments, it appears, that urate of ammonia, when dissolved with about an equal weight of salt, acquires a greater degree of solubility in water, and a difference in appearance from pure urate of ammonia. The appearance is identical with that deposit which can be obtained from urine, and the solubility is more than double the solubility in distilled water. The author tried what effect the salt would have on pure uric acid. He found one part of uric acid remained in 8·941 parts of water, at 68° F., and one part of uric acid, with salt, remained in 7·199

parts of water at 64° F. These results tend to establish Dr. Prout's opinion, by showing how urate of ammonia is modified in form and solubility. The experiments made by Dr. Jones may give a further insight into the various cause of that frequent deposit of urate of ammonia which occurs in health. A small quantity of salt increases the solubility of this substance ; the muriate, the sulphate, and the acetate of ammonia, lessen the dissolving power of distilled water. He concludes, " it is most probable, that each salt that occurs in the urine has some effect on the solubility of the urate of ammonia; and it may be by a very extended inquiry into the relative re-action of the different salts, more particularly the phosphates and sulphates, that we may arrive at an accurate knowledge of the causes of the frequent deposit of urate of ammonia in the urine."

### XIII. CARCINOMA OF THE LUNGS. By *George Burrows*, M.D.

Carcinoma in any part of the body is a terrible disease. In the mamma, the uterus, even in the lip, it is a horrible affliction. In the lungs, and especially in its open state, it is the most direful of all. A few years ago we attended a young married lady, who laboured under this disease. The breath and the expectoration emitted such a malaria, that it was scarcely possible to stay a quarter of an hour in the same room with her, and the effluvium was so dreadful to herself, that she twice attempted suicide ! The odour of cancer is so peculiar that no person can mistake it. It differs totally from that horrible stench attendant on gangrene of the lung, and is, alas ! much more lasting before life becomes extinct. This lady laboured under the malady for years before death terminated her sufferings.

The case which Dr. Burrows relates, did not, apparently, arrive at the state of open cancer, and did not present the fætid breath to which we have alluded. It was a young married female, aged 20 years, who entered St. Bartholomew's Hospital 22nd April, 1843. She had been ill only six months. She complained, at first, of pain beneath the sternum, loss of appetite, cough, and some expectoration, followed by want of sleep, emaciation, and perspiration. A month previous to admission, had an attack of hæmoptysis, succeeded by a pink-coloured sputum. She suckled a healthy child three months old. On admission, she presented the following phenomena :—

"The face pallid, rather full and œdematous, with a dark areola around the eyes; the lips rather livid; the alæ nasi acting violently with each inspiration; respirations 40 in a minute; the pulse 132, rather small, bounding, but soft, and increased to 160 when she assumed the sitting posture in bed; the decubitus on the back, but inclining to the right side.

" She complains of weakness, pain between the shoulders, and gnawing pain in the epigastrium ; also of shortness of breath and of frequent prolonged paroxysms of ineffectual cough, which are followed by urgent dyspnœa amounting to panting; the sputa are scanty, glairy, intimately blended with blood, and of a uniform pink colour, resembling currant-juice ; the glandulæ concatenatæ on the right side of the neck are swollen, hard and tender, with some distended veins passing over them. The glands on the left side of the neck are also slightly enlarged, and the left external jugular vein distended. The tongue clean and moist, the abdomen full, soft and rather tender on pressure in the umbilical region; the bowels open twice daily; the catamenia had not appeared since parturition." 122.

On auscultation, a clear exaggerated respiration, with an increased resonance on percussion, were audible over the left lung—on the other side, a diminished resonance in the upper part; while below the third rib, in front, and beneath the spine of the scapula, there was complete dulness—this dulness extending down to the right hypochondrium. There was a feeble respiratory murmur in the upper part of the right lung. The heart's sounds were natural. The diagnosis was, that she laboured under "extensive malignant disease of the right lung."

We need not follow the details. She died on the fifteenth day after admission.

"The right pleura was distended by Oiv. of an olive brown coloured serum. In spite of this large collection of fluid, the right lung had not collapsed, but stood out firm and prominent into the pleural cavity. The upper lobe of the lung was not much altered: its substance was tough and crepitating on pressure, the middle and lower lobes when handled felt solid. A white lobulated tumor of a dull white colour, something like a mass of suet, projected from the middle lobe of the lung; it was somewhat yielding on pressure, and in close apposition with the right side of the pericardium. Towards the root of this lung was another similar tumor, which forced the lung upwards from the spinal column. The middle lobe was intimately connected with these tumors, and much resembled them in external appearance. The pleura covering the lower lobe was rough and dark-coloured, with enlarged, congested, varicose blood-vessels, ramifying on the surface.

"When sections of the middle lobe and tumors were made, they appeared one continuous mass of carcinoma. Their substance was mostly of an uniform dull white colour, and rather soft; in some parts the substance was pinkish or red, as if vascular, and in other points, especially in the situation of the bronchial glands, the cut surfaces were streaked with black lines and spots, and divided into oval segments. The surfaces yielded on compression a white creamy fluid in considerable quantity." 127.

The diagnosis formed at the beginning, was, if no good luck occurred, one of those extreme instances of auscultic science, which happen once in a century, and to one in a thousand practitioners. We would not advise the tyros of the profession to stake their diagnostic knowledge every day on such minute distinctions. It is only by the "*tactus eruditus*" and the exquisite ear of a master in the art of percussion and auscultation, that a man can hope to predict with the accuracy presented in the foregoing case. We agree with the talented author, that when such a malignant disease as the above is detected during life, the exhibition of mercury, long repeated counter-irritation, frequent blood-letting, &c. "can only impair the vital powers, without arresting the local complaint." But is not the same reasoning to be applied to almost every disease so interfering with the functions of respiration? Can we cure old-standing consolidation or tubercular infiltration of the lungs by medicine? We fear not. "*Optima hic est medicina, medicinam non facere.*"

XIV. CASES OF ACUTE DISEASE IN THE THROAT AND LARYNX. By Dr. *James Arthur Wilson,* Physician to St. George's Hospital.

There can be no doubt that many lives are lost by the above inflammations for want of tracheotomy. In Nov. 1830, Dr. Wilson, with Dr. Nevenson and Mr. Keate, attended a gentleman who died of cynanche supervening on erysipelas. On examination, the epiglottis and posterior membrane of the tongue were found to be highly vascular and thickened, and pus was infiltrated in the cellular membrane of the fauces. The larynx, below the cordæ, vocales, and the trachea were free from disease or obstruction. Here was a case where tracheotomy would have saved life, almost to a certainty. The event made a deep impression on Dr. W.'s mind, and was of service thirteen years afterwards.

*Case.*—Mr. W. C. aged 27, full habit, got heated at a ball, and caught cold going home. He was unable to sleep, from general uneasiness and sense of choking on attempting to swallow. Leeches, calomel, and other measures, were employed; but the breathing was not relieved, even by the abstraction of twenty-four ounces of blood from the arm. In the evening of July 8, 1843, he was in extremis, and Mr. Keate exposed the trachea below the thyroid gland, and made an opening into it, inserting a canula in the aperture. Instantaneous relief was the consequence.

"On the first rush of air into the trachea, the patient appeared to feel instant relief, and his countenance began at once to assume its natural expression; but from this time not two minutes could have elapsed, when he was suddenly attacked by most violent spasms of his whole frame, with a struggle for breath, as if threatening immediate suffocation. All consciousness directly ceased, the eyelids closed, the face was livid, the features were distorted, the blood, still bubbling from the wound, became suddenly black as ink. The breath was drawn convulsively, and at long intervals. All movement, excepting that of the pulse, had ceased, and the patient appeared, literally, at his last gasp. During this awful crisis of the young man's fate, which lasted for perhaps a minute, (seemingly for a much longer time,) his head was held forcibly back,—the canula was withdrawn,—and the orifice in the trachea cleared from blood, and kept widely open. The breathing at length became more natural; the face, no longer ghastly, began to resume the character and tint of life. Not long after this most fearful convulsion, a large quantity of mucus, mixed in part with blood, was rejected, in long viscid ropes, from the mouth; and it was then found that the patient again breathed through the larynx. Upon this, the canula was finally withdrawn. A profuse perspiration now burst forth from the face, neck, and chest of the patient, who gradually recovered his consciousness, and expressed by writing that his 'breathing was quite easy.' He slept at intervals during the night, and was convalescent from this time." 140.

Although, as Dr. Wilson remarks, the operation can hardly be too late, yet the chance of success is greatly lessened by delay, because the patient is being poisoned by his own blood. There is a good deal of management necessary in preventing the blood from flowing down the trachea into the lungs. The operation of tracheotomy has now been so often performed with success, that no patient should be allowed to be suffocated by obstruction about the throat, without opening the wind-pipe. A gentleman of our acquaintance breathed more than twenty years through a tube.

### XV. On the Presence of Oxalate of Lime in the Urine. By *Henry Bence Jones*, M.A., Cantab.

The author observes that the appearance of octohedral crystals in the urine appears first to have been described by M. Vigla. Dr. Bird in England, and afterwards M. Donné in France, recognised the very frequent occurrence of such crystals, and by re-actions observed by the microscope, inferred that these crystals were oxalate of lime. A case occurred to Dr. Jones, in which, from the quantity of octohedral crystals passed, he was able to examine the sediment in nearly the usual method. He remarks:

"The states of the system in which octohedral crystals are seen, vary exceedingly. In acute rheumatism and gout, chronic rheumatism, aggravated hypochondriasis and hysteria, and diabetes, I have found such crystals. In one case in which the rheumatism was slight, the influence of diet and exercise on the mixed deposit of urate of ammonia and oxalate of lime was made the subject of experiment. In other cases in which these octohedral crystals occurred, the symptoms were altogether different; irritation of the urinary organs being the most prominent. The concretion of the crystals, into oxalate of lime-gravel, seemed in one patient to be the cause of this diversity of symptoms.

"The connection observed by MM. Donné and Rayer, between seminal weakness and oxalate of lime, I found in two cases; and in a third, where three small oxalate of lime calculi had been passed at long intervals, and octohedral crystals were constantly found in the urine, on one examination a few dead spermatozoa were seen." 147.

John Saunders, æt. 47, formerly a soldier, was admitted an out-patient of St. George's Hospital. The deposit which had been continually observed in the urine was examined in March 1843, and found to consist of innumerable crystals of uric acid mixed with octohedral crystals. For twenty years he had suffered more or less from urinary disorders. In 1828 he had rheumatic fever, and was confined to bed eleven weeks. The small joints of the fingers are larger and stiffer than natural, and occasionally very painful.

"The urinary sediment was thrown on a filter and washed with distilled water. The red residue was dried, reduced to a fine powder, and treated with dilute hydrochloric acid, which left most of the uric acid undissolved. The acid liquid was filtered, and ammonia gave a very considerable precipitate, when added in excess. When evaporated to dryness, and heated on platinum, the muriate of ammonia was driven off, and the residue effervesced strongly when thrown into dilute acid, and left an alkaline ash when heated more highly. The ash was with difficulty soluble in water, and gave a precipitate with oxalate of ammonia. Hence some organic acid salt of lime was present; and as oxalate of lime is known to occur in octohedral crystals, the conclusion that these crystals were oxalate of lime is most probable." 148.

The author examined the urine of a patient of Mr. Cutler's, and at the same time three small renal calculi which passed in July, August, and September, and afterwards another which passed in October. The urine under the microscope contained multitudes of octohedræ mixed with some crystals of uric acid. All the calculi were found to consist of oxalate of lime mixed with uric acid. He examined the urine in cases of acute rheumatism, and always detected the presence of these octohedral crystals. This deposit is also frequently found mixed with urate of ammonia in chronic rheumatism. In one case he was enabled to make some experiments regarding the effect of diet and exercise on the deposit, and he observed that the octohedral crystals seemed to vary in quantity at different periods of the day. Then follow the daily results of this experiment, which are given very minutely for the four weeks during which it lasted. The author states that it would be easy to multiply examples of the connection between octohedral crystals and rheumatism; but, as no variation in the treatment of ordinary rheumatism seems to be thereby indicated, the fact seems only interesting, as showing the close connection between the red deposit and octohedral crystals. He adds, "octohedral crystals in the urine, and symptoms of a totally different kind frequently occur together. The patient complains of pain in one or both loins, of frequent desire to pass his water, which is sometimes in very small quantity; at other times so much as to simulate diabetes. There are sudden calls to empty the bladder, and if it is delayed, considerable pain is produced. The urine when examined contains only a slight cloud, which does not disappear with heat. In other respects is appears natural. When examined with the microscope, the cloud is seen to consist sometimes entirely of octohedral crystals. More frequently of these crystals mixed with globules of mucus, and sometimes with large and small scales of epithelium."

The above symptoms closely resemble those produced by a small calculus in the kidney, and in one case they suddenly ceased after sharp pain in the course of the right ureter, and slight retraction of the testicle.

Dr. Jones concludes by remarking, that the treatment which proved most beneficial in these cases of irritation was that which improved the general health. In two of Mr. Cutler's patients the symptoms followed mental anxiety. Medicines had little effect, but as the causes for anxiety disappeared, the symptoms ceased.

This paper is a valuable contribution to our knowledge of urinary disorders. We shall return to this important volume in our next number.

